# Supplementary material for: Wild-type Caenorhabditis elegans isolates exhibit distinct gene expression profiles in response to microbial infection
Source: BMC Genomics. 2022 Mar 23;23:229. doi: 10.1186/s12864-022-08455-2 (PMC8943956; doi:10.1186/s12864-022-08455-2)
Supplement: Supplementary file 1 — Additional file 1. [file 12864_2022_8455_MOESM1_ESM.docx]

Supplemental File 1 – *C. elegans* Transcription Factors

| **WormBase Gene ID** | **Gene Name** | **Sequence Name** |
| --- | --- | --- |
| WBGene00000095 | aha-1 | C25A1.11 |
| WBGene00000096 | ahr-1 | C41G7.5 |
| WBGene00000120 | aly-1 | C01F6.5 |
| WBGene00000121 | aly-2 | F23B2.6 |
| WBGene00000220 | atf-2 | K08F8.2 |
| WBGene00000221 | atf-5 | T04C10.4 |
| WBGene00000222 | atf-6 | F45E6.2 |
| WBGene00000223 | atf-7 | C07G2.2 |
| WBGene00000271 | brf-1 | F45E12.2 |
| WBGene00000272 | bro-1 | F56A3.5 |
| WBGene00000366 | cbp-1 | R10E11.1 |
| WBGene00000383 | cdc-14 | C17G10.4 |
| WBGene00000408 | cdk-7 | Y39G10AL.3 |
| WBGene00000428 | ceh-1 | F16H11.4 |
| WBGene00000429 | ceh-2 | C27A12.5 |
| WBGene00000430 | ceh-5 | C16C2.1 |
| WBGene00000431 | ceh-6 | K02B12.1 |
| WBGene00000432 | ceh-7 | C34C6.8 |
| WBGene00000433 | ceh-8 | ZK265.4 |
| WBGene00000434 | ceh-9 | Y65B4BR.9 |
| WBGene00000435 | ceh-10 | W03A3.1 |
| WBGene00000436 | ceh-12 | F33D11.4 |
| WBGene00000437 | ceh-13 | R13A5.5 |
| WBGene00000438 | ceh-14 | F46C8.5 |
| WBGene00000439 | ceh-16 | C13G5.1 |
| WBGene00000440 | ceh-17 | D1007.1 |
| WBGene00000441 | ceh-18 | ZC64.3 |
| WBGene00000442 | ceh-19 | F20D12.6 |
| WBGene00000443 | ceh-20 | F31E3.1 |
| WBGene00000444 | ceh-21 | T26C11.6 |
| WBGene00000445 | ceh-22 | F29F11.5 |
| WBGene00000446 | ceh-23 | ZK652.5 |
| WBGene00000447 | ceh-24 | F55B12.1 |
| WBGene00000448 | pros-1 | K12H4.1 |
| WBGene00000449 | ceh-27 | F46F3.1 |
| WBGene00000450 | ceh-28 | K03A11.3 |
| WBGene00000451 | ceh-30 | C33D12.7 |
| WBGene00000452 | ceh-31 | C33D12.1 |
| WBGene00000453 | ceh-32 | W05E10.3 |
| WBGene00000454 | ceh-33 | C10G8.7 |
| WBGene00000455 | ceh-34 | C10G8.6 |
| WBGene00000457 | ceh-36 | C37E2.4 |
| WBGene00000458 | ceh-37 | C37E2.5 |
| WBGene00000459 | ceh-38 | F22D3.1 |
| WBGene00000460 | ceh-39 | T26C11.7 |
| WBGene00000461 | ceh-40 | F17A2.5 |
| WBGene00000462 | ceh-41 | T26C11.5 |
| WBGene00000463 | ceh-43 | C28A5.4 |
| WBGene00000464 | ceh-44 | Y54F10AM.4 |
| WBGene00000467 | cep-1 | F52B5.5 |
| WBGene00000468 | ces-1 | F43G9.11 |
| WBGene00000469 | ces-2 | ZK909.4 |
| WBGene00000472 | cey-1 | F33A8.3 |
| WBGene00000473 | cey-2 | F46F11.2 |
| WBGene00000474 | cey-3 | M01E11.5 |
| WBGene00000475 | cey-4 | Y39A1C.3 |
| WBGene00000476 | cfi-1 | T23D8.8 |
| WBGene00000483 | che-1 | C55B7.12 |
| WBGene00000521 | cky-1 | C15C8.2 |
| WBGene00000561 | cnd-1 | C34E10.7 |
| WBGene00000584 | cog-1 | R03C1.3 |
| WBGene00000793 | crh-1 | Y41C4A.4 |
| WBGene00000895 | dac-1 | B0412.1 |
| WBGene00000899 | daf-3 | F25E2.5 |
| WBGene00000901 | daf-5 | W01G7.1 |
| WBGene00000904 | daf-8 | R05D11.1 |
| WBGene00000908 | daf-12 | F11A1.3 |
| WBGene00000910 | daf-14 | F01G10.8 |
| WBGene00000912 | daf-16 | R13H8.1 |
| WBGene00000914 | daf-19 | F33H1.1 |
| WBGene00000936 | dbl-1 | T25F10.2 |
| WBGene00000938 | dcp-66 | C26C6.5 |
| WBGene00000995 | die-1 | C18D1.1 |
| WBGene00001029 | dnj-11 | F38A5.13 |
| WBGene00001035 | dnj-17 | T03F6.2 |
| WBGene00001061 | dpl-1 | T23G7.1 |
| WBGene00001062 | dpr-1 | R10D12.2 |
| WBGene00001079 | dpy-20 | T22B3.1 |
| WBGene00001081 | dpy-22 | F47A4.2 |
| WBGene00001092 | dro-1 | F53A2.5 |
| WBGene00001096 | dsc-1 | C18B12.3 |
| WBGene00001159 | eff-1 | C26D10.5 |
| WBGene00001161 | efl-1 | Y102A5C.18 |
| WBGene00001162 | efl-2 | Y48C3A.17 |
| WBGene00001174 | egl-5 | C08C3.1 |
| WBGene00001182 | egl-13 | T22B7.1 |
| WBGene00001186 | egl-18 | F55A8.1 |
| WBGene00001194 | egl-27 | C04A2.3 |
| WBGene00001204 | egl-38 | C04G2.7 |
| WBGene00001207 | egl-43 | R53.3 |
| WBGene00001208 | egl-44 | F28B12.2 |
| WBGene00001210 | egl-46 | K11G9.4 |
| WBGene00001223 | ehn-3 | ZK616.10 |
| WBGene00001235 | elb-1 | Y41C4A.10 |
| WBGene00001249 | elt-1 | W09C2.1 |
| WBGene00001250 | elt-2 | C33D3.1 |
| WBGene00001251 | elt-3 | K02B9.4 |
| WBGene00001252 | elt-4 | C39B10.6 |
| WBGene00001253 | elt-6 | F52C12.5 |
| WBGene00001259 | emb-5 | T04A8.14 |
| WBGene00001310 | end-1 | F58E10.2 |
| WBGene00001311 | end-3 | F58E10.5 |
| WBGene00001324 | eor-1 | R11E3.6 |
| WBGene00001345 | fos-1 | F29G9.4 |
| WBGene00001400 | fax-1 | F56E3.4 |
| WBGene00001434 | fkh-2 | T14G12.4 |
| WBGene00001435 | fkh-3 | C29F7.4 |
| WBGene00001436 | fkh-4 | C29F7.5 |
| WBGene00001437 | fkh-5 | F26A1.2 |
| WBGene00001438 | fkh-6 | B0286.5 |
| WBGene00001439 | fkh-7 | F26D12.1 |
| WBGene00001440 | fkh-8 | F40H3.4 |
| WBGene00001441 | fkh-9 | K03C7.2 |
| WBGene00001442 | fkh-10 | C25A1.2 |
| WBGene00001493 | frm-7 | C51F7.1 |
| WBGene00001514 | xnd-1 | C05D2.5 |
| WBGene00001560 | gei-3 | T22H6.6 |
| WBGene00001565 | gei-8 | C14B9.6 |
| WBGene00001568 | snpc-4 | F32H2.1 |
| WBGene00001570 | gei-13 | F58A4.11 |
| WBGene00001574 | gei-17 | W10D5.3 |
| WBGene00001707 | grh-1 | Y48G8AR.1 |
| WBGene00001820 | ham-1 | F53B2.6 |
| WBGene00001821 | ham-2 | C07A12.1 |
| WBGene00001824 | hbl-1 | F13D11.2 |
| WBGene00001827 | hcf-1 | C46A5.9 |
| WBGene00001851 | hif-1 | F38A6.3 |
| WBGene00001867 | him-8 | T07G12.12 |
| WBGene00001948 | hlh-1 | B0304.1 |
| WBGene00001949 | hlh-2 | M05B5.5 |
| WBGene00001950 | hlh-3 | T24B8.6 |
| WBGene00001951 | hlh-4 | T05G5.2 |
| WBGene00001952 | hlh-6 | T15H9.3 |
| WBGene00001953 | hlh-8 | C02B8.4 |
| WBGene00001954 | hlh-10 | ZK682.4 |
| WBGene00001955 | hlh-11 | F58A4.7 |
| WBGene00001956 | hlh-12 | C28C12.8 |
| WBGene00001957 | hlh-13 | F48D6.3 |
| WBGene00001958 | hlh-14 | C18A3.8 |
| WBGene00001959 | hlh-15 | C43H6.8 |
| WBGene00001960 | hlh-16 | DY3.3 |
| WBGene00001961 | hlh-17 | F38C2.2 |
| WBGene00001962 | hlh-19 | F57C12.3 |
| WBGene00001964 | hlh-25 | C17C3.7 |
| WBGene00001965 | hlh-26 | C17C3.8 |
| WBGene00001966 | hlh-27 | C17C3.10 |
| WBGene00001967 | hlh-28 | F31A3.2 |
| WBGene00001968 | hlh-29 | F31A3.4 |
| WBGene00001971 | hmg-1.1 | Y48B6A.14 |
| WBGene00001972 | hmg-1.2 | F47D12.4 |
| WBGene00001973 | hmg-3 | C32F10.5 |
| WBGene00001974 | hmg-4 | T20B12.8 |
| WBGene00001975 | hmg-5 | F45E4.9 |
| WBGene00001976 | hmg-11 | T05A7.4 |
| WBGene00001977 | hmg-12 | Y17G7A.1 |
| WBGene00001981 | hnd-1 | C44C10.8 |
| WBGene00002002 | hsb-1 | K08E7.2 |
| WBGene00002004 | hsf-1 | Y53C10A.12 |
| WBGene00002025 | hsp-60 | Y22D7AL.5 |
| WBGene00002045 | icd-1 | C56C10.8 |
| WBGene00002169 | isw-1 | F37A4.8 |
| WBGene00002245 | lag-1 | K08B4.1 |
| WBGene00002261 | ldb-1 | F58A3.1 |
| WBGene00002601 | let-381 | F26B1.7 |
| WBGene00002717 | let-526 | C01G8.9 |
| WBGene00002783 | let-607 | F57B10.1 |
| WBGene00002987 | lim-4 | ZC64.4 |
| WBGene00002988 | lim-6 | K03E6.1 |
| WBGene00002989 | lim-7 | C04F1.3 |
| WBGene00002990 | lin-1 | C37F5.1 |
| WBGene00003000 | lin-11 | ZC247.3 |
| WBGene00003002 | lin-13 | C03B8.4 |
| WBGene00003008 | lin-22 | Y54G2A.1 |
| WBGene00003012 | lin-26 | F18A1.2 |
| WBGene00003014 | lin-28 | F02E9.2 |
| WBGene00003015 | lin-29 | W03C9.4 |
| WBGene00003017 | lin-31 | K10G6.1 |
| WBGene00003018 | lin-32 | T14F9.5 |
| WBGene00003021 | lin-36 | F44B9.6 |
| WBGene00003024 | lin-39 | C07H6.7 |
| WBGene00003025 | lin-40 | T27C4.4 |
| WBGene00003033 | lin-48 | F34D10.5 |
| WBGene00003037 | lin-54 | JC8.6 |
| WBGene00003044 | lir-1 | F18A1.3 |
| WBGene00003045 | lir-2 | F18A1.4 |
| WBGene00003046 | lir-3 | F37H8.1 |
| WBGene00003059 | lpd-2 | C48E7.3 |
| WBGene00003087 | lsy-2 | F49H12.1 |
| WBGene00003100 | mab-3 | Y53C12B.5 |
| WBGene00003102 | mab-5 | C08C3.3 |
| WBGene00003106 | mab-9 | T27A1.6 |
| WBGene00003107 | mab-10 | R166.1 |
| WBGene00003114 | mab-23 | C32C4.5 |
| WBGene00003148 | mbf-1 | H21P03.1 |
| WBGene00003163 | mdl-1 | R03E9.1 |
| WBGene00003167 | mec-3 | F01D4.6 |
| WBGene00003180 | med-1 | T24D3.1 |
| WBGene00003181 | med-2 | K04C2.6 |
| WBGene00003182 | mef-2 | W10D5.1 |
| WBGene00003210 | mel-28 | C38D4.3 |
| WBGene00003218 | mep-1 | M04B2.1 |
| WBGene00003228 | mex-1 | W03C9.7 |
| WBGene00003230 | mex-5 | W02A2.7 |
| WBGene00003231 | mex-6 | AH6.5 |
| WBGene00003233 | mgl-2 | F45H11.4 |
| WBGene00003241 | mig-5 | T05C12.6 |
| WBGene00003376 | mls-1 | H14A12.4 |
| WBGene00003377 | mls-2 | C39E6.4 |
| WBGene00003378 | mml-1 | T20B12.6 |
| WBGene00003380 | mnm-2 | C10A4.8 |
| WBGene00003388 | moe-3 | F32A11.6 |
| WBGene00003406 | mrg-1 | Y37D8A.9 |
| WBGene00003480 | klf-3 | F54H5.4 |
| WBGene00003509 | mxl-1 | T19B10.11 |
| WBGene00003510 | mxl-2 | F40G9.11 |
| WBGene00003511 | mxl-3 | F46G10.6 |
| WBGene00003592 | nfi-1 | ZK1290.4 |
| WBGene00003595 | ngn-1 | Y69A2AR.29 |
| WBGene00003600 | nhr-1 | R09G11.2 |
| WBGene00003601 | nhr-2 | C32F10.6 |
| WBGene00003602 | nhr-3 | H01A20.1 |
| WBGene00003603 | nhr-4 | F32B6.1 |
| WBGene00003604 | nhr-5 | Y73F8A.21 |
| WBGene00003605 | nhr-6 | C48D5.1 |
| WBGene00003606 | nhr-7 | F54D1.4 |
| WBGene00003607 | nhr-8 | F33D4.1 |
| WBGene00003608 | nhr-9 | ZK418.1 |
| WBGene00003609 | nhr-10 | B0280.8 |
| WBGene00003610 | nhr-11 | ZC410.1 |
| WBGene00003611 | nhr-12 | R04B5.4 |
| WBGene00003612 | nhr-13 | Y5H2B.2 |
| WBGene00003613 | nhr-14 | T01B10.4 |
| WBGene00003614 | nhr-15 | F33E11.1 |
| WBGene00003615 | nhr-16 | T12C9.6 |
| WBGene00003616 | nhr-17 | C02B4.2 |
| WBGene00003617 | nhr-18 | F44C8.3 |
| WBGene00003618 | nhr-19 | E02H1.7 |
| WBGene00003619 | nhr-20 | F43C1.4 |
| WBGene00003620 | nhr-21 | F21D12.1 |
| WBGene00003621 | nhr-22 | K06A1.4 |
| WBGene00003622 | nhr-23 | C01H6.5 |
| WBGene00003623 | nhr-25 | F11C1.6 |
| WBGene00003624 | nhr-28 | C11G6.4 |
| WBGene00003625 | nhr-31 | C26B2.3 |
| WBGene00003626 | nhr-32 | K08H2.8 |
| WBGene00003627 | nhr-34 | F58G6.5 |
| WBGene00003628 | nhr-35 | C07A12.3 |
| WBGene00003629 | nhr-38 | K01H12.3 |
| WBGene00003630 | nhr-40 | T03G6.2 |
| WBGene00003632 | nhr-42 | C33G8.6 |
| WBGene00003633 | nhr-43 | C29E6.5 |
| WBGene00003634 | nhr-44 | T19A5.4 |
| WBGene00003635 | nhr-45 | F16H11.5 |
| WBGene00003636 | nhr-46 | C45E5.6 |
| WBGene00003637 | nhr-47 | C24G6.4 |
| WBGene00003638 | nhr-48 | ZK662.3 |
| WBGene00003639 | nhr-49 | K10C3.6 |
| WBGene00003640 | nhr-50 | C06C6.5 |
| WBGene00003641 | nhr-51 | K06B4.1 |
| WBGene00003642 | nhr-52 | K06B4.2 |
| WBGene00003643 | nhr-53 | K06B4.11 |
| WBGene00003644 | nhr-54 | F36D3.2 |
| WBGene00003645 | nhr-55 | T01G6.7 |
| WBGene00003646 | nhr-56 | F44C8.6 |
| WBGene00003647 | nhr-57 | T05B4.2 |
| WBGene00003648 | nhr-58 | R11G11.2 |
| WBGene00003649 | nhr-59 | T27B7.1 |
| WBGene00003650 | nhr-60 | F57A10.5 |
| WBGene00003651 | nhr-61 | W01D2.2 |
| WBGene00003652 | nhr-62 | Y67A6A.2 |
| WBGene00003653 | nhr-63 | C06C6.4 |
| WBGene00003654 | nhr-64 | C45E1.1 |
| WBGene00003655 | nhr-65 | Y17D7A.3 |
| WBGene00003656 | nhr-66 | T09A12.4 |
| WBGene00003657 | nhr-67 | C08F8.8 |
| WBGene00003658 | nhr-68 | H12C20.3 |
| WBGene00003659 | nhr-69 | T23H4.2 |
| WBGene00003660 | nhr-70 | Y51A2D.17 |
| WBGene00003661 | nhr-71 | K11E4.5 |
| WBGene00003662 | nhr-72 | C17A2.8 |
| WBGene00003663 | nhr-73 | C27C7.4 |
| WBGene00003664 | nhr-74 | C27C7.3 |
| WBGene00003665 | nhr-75 | C49D10.6 |
| WBGene00003667 | nhr-77 | T15D6.6 |
| WBGene00003668 | nhr-78 | F36A4.14 |
| WBGene00003669 | nhr-79 | T26H2.9 |
| WBGene00003670 | nhr-80 | H10E21.3 |
| WBGene00003671 | nhr-81 | C47F8.8 |
| WBGene00003672 | nhr-82 | F41D3.1 |
| WBGene00003673 | nhr-83 | F48G7.3 |
| WBGene00003674 | nhr-84 | T06C12.7 |
| WBGene00003675 | nhr-85 | W05B5.3 |
| WBGene00003676 | nhr-86 | Y40B10A.8 |
| WBGene00003677 | nhr-87 | Y41D4B.7 |
| WBGene00003678 | nhr-88 | K08A2.5 |
| WBGene00003679 | nhr-89 | E03H4.13 |
| WBGene00003680 | nhr-90 | ZK488.2 |
| WBGene00003681 | nhr-91 | Y15E3A.1 |
| WBGene00003682 | nhr-92 | Y41D4B.8 |
| WBGene00003683 | nhr-93 | T24A6.9 |
| WBGene00003684 | nhr-94 | C12D5.8 |
| WBGene00003685 | nhr-95 | Y39B6A.17 |
| WBGene00003686 | nhr-96 | F44C8.11 |
| WBGene00003687 | nhr-97 | H27C11.1 |
| WBGene00003688 | nhr-98 | M02H5.6 |
| WBGene00003689 | nhr-99 | M02H5.1 |
| WBGene00003690 | nhr-100 | C28D4.1 |
| WBGene00003691 | nhr-101 | H12C20.6 |
| WBGene00003692 | nhr-102 | T06C12.6 |
| WBGene00003693 | nhr-103 | F44C8.4 |
| WBGene00003694 | nhr-104 | R11E3.5 |
| WBGene00003695 | nhr-105 | C06G3.1 |
| WBGene00003696 | nhr-106 | T01G6.4 |
| WBGene00003697 | nhr-107 | C33G8.11 |
| WBGene00003698 | nhr-108 | F35E8.12 |
| WBGene00003699 | nhr-109 | T12C9.5 |
| WBGene00003700 | nhr-110 | Y46H3D.5 |
| WBGene00003701 | nhr-111 | F44G3.9 |
| WBGene00003702 | nhr-112 | Y70C5C.6 |
| WBGene00003703 | nhr-113 | ZK1025.9 |
| WBGene00003704 | nhr-114 | Y45G5AM.1 |
| WBGene00003705 | nhr-115 | T27B7.4 |
| WBGene00003706 | nhr-116 | F09C6.9 |
| WBGene00003707 | nhr-117 | F16B4.12 |
| WBGene00003708 | nhr-118 | F13A2.8 |
| WBGene00003709 | nhr-119 | K12H6.1 |
| WBGene00003710 | nhr-120 | C25B8.6 |
| WBGene00003711 | nhr-121 | E02H9.8 |
| WBGene00003712 | nhr-122 | Y41D4B.9 |
| WBGene00003713 | nhr-123 | M02H5.7 |
| WBGene00003714 | nhr-124 | C17E7.8 |
| WBGene00003715 | nhr-125 | R02D1.1 |
| WBGene00003716 | nhr-126 | F44C8.10 |
| WBGene00003717 | nhr-127 | T13F3.3 |
| WBGene00003718 | nhr-128 | F44C8.5 |
| WBGene00003719 | nhr-129 | C50B6.14 |
| WBGene00003720 | nhr-130 | T01G6.8 |
| WBGene00003721 | nhr-131 | T01G6.2 |
| WBGene00003722 | nhr-132 | R11G11.1 |
| WBGene00003723 | nhr-133 | F44C8.8 |
| WBGene00003724 | nhr-134 | F44C8.2 |
| WBGene00003725 | nhr-135 | VC5.5 |
| WBGene00003726 | nhr-136 | C13C4.3 |
| WBGene00003727 | nhr-137 | C56E10.4 |
| WBGene00003728 | nhr-138 | C28D4.9 |
| WBGene00003779 | nob-1 | Y75B8A.2 |
| WBGene00003834 | nxf-1 | C15H11.3 |
| WBGene00003845 | odd-1 | B0280.4 |
| WBGene00003846 | odd-2 | C34H3.2 |
| WBGene00003847 | blmp-1 | F25D7.3 |
| WBGene00003854 | odr-7 | T18D3.2 |
| WBGene00003864 | oma-1 | C09G9.6 |
| WBGene00003865 | oma-2 | ZC513.6 |
| WBGene00003909 | pag-3 | F45B8.4 |
| WBGene00003912 | pal-1 | C38D4.6 |
| WBGene00003933 | pat-9 | T27B1.2 |
| WBGene00003937 | pax-1 | K07C11.1 |
| WBGene00003938 | pax-2 | K06B9.5 |
| WBGene00003939 | pax-3 | F27E5.2 |
| WBGene00003968 | peb-1 | T14F9.4 |
| WBGene00003976 | pes-1 | T28H11.4 |
| WBGene00004011 | pha-2 | M6.3 |
| WBGene00004013 | pha-4 | F38A6.1 |
| WBGene00004024 | php-3 | Y75B8A.1 |
| WBGene00004027 | pie-1 | Y49E10.14 |
| WBGene00004046 | plp-1 | F45E4.2 |
| WBGene00004048 | plx-2 | K04B12.1 |
| WBGene00004077 | pop-1 | W10C8.2 |
| WBGene00004078 | pos-1 | F52E1.1 |
| WBGene00004096 | pqm-1 | F40F8.7 |
| WBGene00004106 | pqn-15 | C24A8.3 |
| WBGene00004111 | pqn-21 | C37A2.5 |
| WBGene00004134 | myrf-1 | F59B10.1 |
| WBGene00004136 | pqn-51 | K11D12.2 |
| WBGene00004157 | pqn-75 | W03D2.1 |
| WBGene00004203 | swsn-1 | Y113G7B.23 |
| WBGene00004204 | swsn-4 | F01G4.1 |
| WBGene00004319 | rbr-2 | ZK593.4 |
| WBGene00004334 | ref-1 | T01E8.2 |
| WBGene00004335 | ref-2 | C47C12.3 |
| WBGene00004393 | rnt-1 | B0414.2 |
| WBGene00004703 | rsp-6 | C33H5.12 |
| WBGene00004735 | sbp-1 | Y47D3B.7 |
| WBGene00004745 | sdc-1 | F52E10.1 |
| WBGene00004747 | sdc-3 | C25D7.3 |
| WBGene00004750 | sea-1 | F19B10.9 |
| WBGene00004751 | sea-2 | K10G6.3 |
| WBGene00004771 | sem-2 | C32E12.5 |
| WBGene00004773 | sem-4 | F15C11.1 |
| WBGene00004786 | sex-1 | F44A6.2 |
| WBGene00004803 | sir-2.4 | C06A5.11 |
| WBGene00004804 | skn-1 | T19E7.2 |
| WBGene00004856 | sma-2 | ZK370.2 |
| WBGene00004857 | sma-3 | R13F6.9 |
| WBGene00004858 | sma-4 | R12B2.1 |
| WBGene00004860 | sma-6 | C32D5.2 |
| WBGene00004862 | sma-9 | T05A10.1 |
| WBGene00004911 | snf-12 | T25B6.7 |
| WBGene00004949 | sox-2 | K08A8.2 |
| WBGene00004950 | sox-3 | F40E10.2 |
| WBGene00004964 | spe-10 | AC3.10 |
| WBGene00005006 | spr-1 | D1014.8 |
| WBGene00005008 | spr-3 | C07A12.5 |
| WBGene00005009 | spr-4 | C09H6.1 |
| WBGene00005011 |  | F26F4.8 |
| WBGene00005013 | jmjd-1.1 | F43G6.6 |
| WBGene00005014 | spt-4 | F54C4.2 |
| WBGene00005015 | spt-5 | K08E4.1 |
| WBGene00006342 | sup-37 | C01B7.1 |
| WBGene00006379 | sys-1 | T23D8.9 |
| WBGene00006380 | tab-1 | F31E8.3 |
| WBGene00006382 | taf-1 | W04A8.7 |
| WBGene00006383 | taf-2 | Y37E11B.4 |
| WBGene00006384 | taf-3 | C11G6.1 |
| WBGene00006385 | taf-4 | R119.6 |
| WBGene00006386 | taf-5 | F30F8.8 |
| WBGene00006387 | taf-6.1 | W09B6.2 |
| WBGene00006388 | taf-7.1 | F54F7.1 |
| WBGene00006389 | taf-7.2 | Y111B2A.16 |
| WBGene00006390 | taf-8 | ZK1320.12 |
| WBGene00006391 | taf-9 | T12D8.7 |
| WBGene00006392 | taf-10 | K03B4.3 |
| WBGene00006393 | taf-11.1 | F48D6.1 |
| WBGene00006394 | taf-11.2 | K10D3.3 |
| WBGene00006395 | taf-11.3 | F43D9.5 |
| WBGene00006396 | taf-12 | Y56A3A.4 |
| WBGene00006397 | taf-13 | C14A4.10 |
| WBGene00006424 | ctbp-1 | F49E10.5 |
| WBGene00006445 | tag-68 | F37D6.6 |
| WBGene00006462 | tag-97 | C33A11.4 |
| WBGene00006471 | nhr-233 | Y32B12B.6 |
| WBGene00006474 | wdr-5.1 | C14B1.4 |
| WBGene00006476 | rhgf-2 | T08H4.1 |
| WBGene00006492 | let-391 | C27A12.3 |
| WBGene00006542 | tbp-1 | T20B12.2 |
| WBGene00006543 | tbx-2 | F21H11.3 |
| WBGene00006544 | tbx-7 | ZK328.8 |
| WBGene00006545 | tbx-8 | T07C4.2 |
| WBGene00006546 | tbx-9 | T07C4.6 |
| WBGene00006547 | tbx-11 | F40H6.4 |
| WBGene00006549 | tbx-30 | Y59E9AR.3 |
| WBGene00006550 | tbx-31 | C36C9.2 |
| WBGene00006551 | tbx-32 | ZK380.1 |
| WBGene00006552 | tbx-33 | Y66A7A.8 |
| WBGene00006553 | tbx-34 | Y47D3A.10 |
| WBGene00006554 | tbx-35 | ZK177.10 |
| WBGene00006555 | tbx-36 | ZK829.5 |
| WBGene00006556 | tbx-37 | Y47D3A.12 |
| WBGene00006557 | tbx-38 | C24H11.3 |
| WBGene00006558 | tbx-39 | Y73F8A.16 |
| WBGene00006559 | tbx-40 | Y73F8A.17 |
| WBGene00006560 | tbx-41 | T26C11.1 |
| WBGene00006577 | tlf-1 | F39H11.2 |
| WBGene00006580 | tlp-1 | T23G4.1 |
| WBGene00006604 | tra-1 | Y47D3A.6 |
| WBGene00006648 | ttb-1 | W03F9.5 |
| WBGene00006652 | ttx-1 | Y113G7A.6 |
| WBGene00006654 | ttx-3 | C40H5.5 |
| WBGene00006698 | uaf-2 | Y116A8C.35 |
| WBGene00006706 | ubc-9 | F29B9.6 |
| WBGene00006726 | ubl-5 | F46F11.4 |
| WBGene00006739 | ulp-4 | C41C4.6 |
| WBGene00006743 | unc-3 | Y16B4A.1 |
| WBGene00006744 | unc-4 | F26C11.2 |
| WBGene00006766 | unc-30 | B0564.10 |
| WBGene00006773 | unc-37 | W02D3.9 |
| WBGene00006775 | unc-39 | F56A12.1 |
| WBGene00006778 | unc-42 | F58E6.10 |
| WBGene00006790 | unc-55 | F55D12.4 |
| WBGene00006796 | unc-62 | T28F12.2 |
| WBGene00006818 | unc-86 | C30A5.7 |
| WBGene00006827 | unc-98 | F08C6.7 |
| WBGene00006844 | unc-120 | D1081.2 |
| WBGene00006853 | unc-130 | C47G2.2 |
| WBGene00006870 | vab-3 | F14F3.1 |
| WBGene00006873 | vab-7 | M142.4 |
| WBGene00006881 | vab-15 | R07B1.1 |
| WBGene00006943 | wrm-1 | B0336.1 |
| WBGene00006959 | xbp-1 | R74.3 |
| WBGene00006970 | zag-1 | F28F9.1 |
| WBGene00006986 | zip-1 | Y75B8A.35 |
| WBGene00007042 | pbrm-1 | C26C6.1 |
| WBGene00007048 | nfx-1 | C16A3.7 |
| WBGene00007053 | chd-7 | T04D1.4 |
| WBGene00007058 | dmd-6 | F13G11.1 |
| WBGene00007063 |  | 2L52.1 |
| WBGene00007094 |  | B0019.2 |
| WBGene00007105 |  | B0035.1 |
| WBGene00007121 |  | B0250.4 |
| WBGene00007195 | thoc-7 | B0513.2 |
| WBGene00007223 |  | C01F6.9 |
| WBGene00007242 | madf-5 | C01G12.1 |
| WBGene00007367 | nhr-150 | C06B8.1 |
| WBGene00007387 | jmjd-5 | C06H2.3 |
| WBGene00007416 | ceh-57 | C07E3.5 |
| WBGene00007417 | ceh-58 | C07E3.6 |
| WBGene00007433 | swsn-7 | C08B11.3 |
| WBGene00007496 | npax-4 | C09G9.7 |
| WBGene00007546 | nhr-153 | C13C4.1 |
| WBGene00007547 | nhr-154 | C13C4.2 |
| WBGene00007625 |  | C16C10.4 |
| WBGene00007637 | dhhc-7 | C17D12.1 |
| WBGene00007645 |  | C17E4.6 |
| WBGene00007732 | spe-44 | C25G4.4 |
| WBGene00007749 | ceh-79 | C26E1.3 |
| WBGene00007761 | rad-26 | C27B7.4 |
| WBGene00007772 | egrh-1 | C27C12.2 |
| WBGene00007776 | dmd-4 | C27C12.6 |
| WBGene00007813 | jmjd-3.3 | C29F7.6 |
| WBGene00007877 | nfki-1 | C33A11.1 |
| WBGene00007907 |  | C34B4.2 |
| WBGene00007929 | dmd-10 | C34D1.1 |
| WBGene00007930 | dmd-11 | C34D1.2 |
| WBGene00007932 | zip-5 | C34D1.5 |
| WBGene00007943 |  | C34F6.9 |
| WBGene00007961 |  | C35D6.4 |
| WBGene00007980 |  | C36E8.1 |
| WBGene00007984 | irx-1 | C36F7.1 |
| WBGene00008007 |  | C38D4.7 |
| WBGene00008056 | nhr-164 | C41G6.5 |
| WBGene00008081 | athp-1 | C44B9.4 |
| WBGene00008092 | gmeb-3 | C44F1.2 |
| WBGene00008118 | madf-8 | C46F11.3 |
| WBGene00008158 | nhr-165 | C47F8.2 |
| WBGene00008195 | ceh-88 | C49C3.5 |
| WBGene00008208 | nhr-167 | C49F5.4 |
| WBGene00008209 |  | C49F5.5 |
| WBGene00008221 | nhr-168 | C50B6.8 |
| WBGene00008242 | ceh-75 | C50H2.6 |
| WBGene00008289 | nhr-169 | C54C8.1 |
| WBGene00008309 | nhr-170 | C54E10.5 |
| WBGene00008338 | pafo-1 | C55A6.9 |
| WBGene00008363 |  | D1046.2 |
| WBGene00008386 | cdc-5L | D1081.8 |
| WBGene00008417 |  | D2030.7 |
| WBGene00008419 | wdr-23 | D2030.9 |
| WBGene00008474 | nhr-174 | E03H4.6 |
| WBGene00008606 | dhhc-1 | F09B12.2 |
| WBGene00008619 | nhr-262 | F09C6.8 |
| WBGene00008630 | nhr-175 | F09F3.10 |
| WBGene00008640 |  | F10B5.3 |
| WBGene00008683 | repo-1 | F11A10.2 |
| WBGene00008748 | yap-1 | F13E6.4 |
| WBGene00008762 | ztf-2 | F13G3.1 |
| WBGene00008778 | nhr-264 | F14A5.1 |
| WBGene00008830 | nhr-176 | F14H3.11 |
| WBGene00008882 |  | F16B12.6 |
| WBGene00008884 | nhr-281 | F16B12.8 |
| WBGene00008901 | nhr-27 | F16H9.2 |
| WBGene00008976 | plp-2 | F20D1.4 |
| WBGene00008999 | myrf-2 | F21A10.2 |
| WBGene00009014 |  | F21D5.9 |
| WBGene00009026 |  | F21G4.5 |
| WBGene00009050 |  | F22D6.2 |
| WBGene00009071 |  | F23A7.6 |
| WBGene00009084 |  | F23B12.7 |
| WBGene00009089 | jmjd-3.2 | F23D12.5 |
| WBGene00009133 | bed-3 | F25H8.6 |
| WBGene00009174 |  | F26H9.2 |
| WBGene00009180 | nurf-1 | F26H11.2 |
| WBGene00009188 | lsy-22 | F27D4.2 |
| WBGene00009189 |  | F27D4.4 |
| WBGene00009190 |  | F27D4.6 |
| WBGene00009202 | aptf-4 | F28C6.1 |
| WBGene00009203 | aptf-3 | F28C6.2 |
| WBGene00009224 |  | F28F8.7 |
| WBGene00009231 | ceh-89 | F28H6.2 |
| WBGene00009341 | thoc-3 | F32H2.4 |
| WBGene00009365 |  | F33H1.4 |
| WBGene00009448 | zfp-2 | F35H8.3 |
| WBGene00009461 | madf-2 | F36D1.1 |
| WBGene00009475 |  | F36F2.1 |
| WBGene00009508 | row-1 | F37D6.2 |
| WBGene00009532 | ccch-1 | F38B7.1 |
| WBGene00009537 | ccch-2 | F38C2.5 |
| WBGene00009539 |  | F38C2.7 |
| WBGene00009540 | hlh-31 | F38C2.8 |
| WBGene00009553 | hinf-1 | F39B2.1 |
| WBGene00009560 | psa-3 | F39D8.2 |
| WBGene00009584 | drap-1 | F40F9.7 |
| WBGene00009608 | nhr-265 | F41D3.3 |
| WBGene00009650 | ccnk-1 | F43D2.1 |
| WBGene00009672 |  | F43G9.12 |
| WBGene00009687 |  | F44D12.10 |
| WBGene00009743 | sptf-1 | F45H11.1 |
| WBGene00009772 | ztf-7 | F46B6.7 |
| WBGene00009827 | hmg-6 | F47G4.6 |
| WBGene00009834 | lsy-27 | F47H4.1 |
| WBGene00009899 | efl-3 | F49E12.6 |
| WBGene00009923 |  | F52B5.7 |
| WBGene00009937 | lsl-1 | F52F12.4 |
| WBGene00009939 | ztf-11 | F52F12.6 |
| WBGene00009965 | frpr-9 | F53B7.2 |
| WBGene00009998 | klf-2 | F53F8.1 |
| WBGene00010010 | gmeb-2 | F53H4.5 |
| WBGene00010012 | saeg-1 | F53H10.2 |
| WBGene00010054 | gtf-2E2 | F54D5.11 |
| WBGene00010086 |  | F55B11.4 |
| WBGene00010137 | ztf-26 | F55H12.6 |
| WBGene00010180 | nhr-192 | F57A8.5 |
| WBGene00010186 | nhr-283 | F57A10.6 |
| WBGene00010215 | nhr-193 | F57G8.6 |
| WBGene00010251 | sta-2 | F58E6.1 |
| WBGene00010264 |  | F58G1.2 |
| WBGene00010323 | dhhc-12 | F59C6.2 |
| WBGene00010401 |  | H16D19.3 |
| WBGene00010406 | math-33 | H19N07.2 |
| WBGene00010410 | nhr-267 | H22D14.1 |
| WBGene00010453 | fozi-1 | K01B6.1 |
| WBGene00010476 | rnf-113 | K01G5.1 |
| WBGene00010553 |  | K04C1.3 |
| WBGene00010600 | nhr-196 | K06B4.5 |
| WBGene00010601 | nhr-268 | K06B4.6 |
| WBGene00010602 | nhr-197 | K06B4.7 |
| WBGene00010603 | nhr-198 | K06B4.8 |
| WBGene00010604 | nhr-199 | K06B4.10 |
| WBGene00010704 |  | K09A11.1 |
| WBGene00010770 |  | K11D2.4 |
| WBGene00010781 |  | K11H3.4 |
| WBGene00010868 | somi-1 | M04G12.4 |
| WBGene00010892 | dhhc-6 | M18.8 |
| WBGene00010936 | ztf-14 | M163.2 |
| WBGene00010990 | tceb-3 | R03D7.4 |
| WBGene00010995 | ceh-90 | R03E1.4 |
| WBGene00011002 | nhr-205 | R04B5.3 |
| WBGene00011066 | ztf-15 | R06C7.9 |
| WBGene00011069 | ceh-62 | R06F6.6 |
| WBGene00011097 | nhr-206 | R07B7.13 |
| WBGene00011098 | nhr-207 | R07B7.14 |
| WBGene00011099 | nhr-208 | R07B7.15 |
| WBGene00011100 | nhr-209 | R07B7.16 |
| WBGene00011113 |  | R07E5.5 |
| WBGene00011130 | zip-6 | R07H5.10 |
| WBGene00011150 | nhr-269 | R08H2.9 |
| WBGene00011206 |  | R10E4.11 |
| WBGene00011257 | npax-3 | R13.2 |
| WBGene00011259 | miz-1 | R13.4 |
| WBGene00011315 | mbr-1 | T01C1.2 |
| WBGene00011319 |  | T01C3.2 |
| WBGene00011327 | hlh-34 | T01D3.2 |
| WBGene00011368 | tftc-5 | T02C12.3 |
| WBGene00011376 | gla-3 | T02E1.3 |
| WBGene00011396 | nhr-271 | T03E6.3 |
| WBGene00011505 | pzf-1 | T05G11.1 |
| WBGene00011520 | nhr-213 | T06C12.13 |
| WBGene00011547 |  | T06G6.5 |
| WBGene00011563 | jmjd-4 | T07C4.11 |
| WBGene00011565 | nhr-272 | T07C5.2 |
| WBGene00011566 | nhr-214 | T07C5.3 |
| WBGene00011567 | nhr-215 | T07C5.4 |
| WBGene00011568 | nhr-26 | T07C5.5 |
| WBGene00011575 | madf-4 | T07C12.11 |
| WBGene00011583 |  | T07D10.3 |
| WBGene00011597 | zim-1 | T07G12.6 |
| WBGene00011600 | zim-2 | T07G12.10 |
| WBGene00011601 | zim-3 | T07G12.11 |
| WBGene00011614 | nfya-1 | T08D10.1 |
| WBGene00011615 | lsd-1 | T08D10.2 |
| WBGene00011626 |  | T08G5.7 |
| WBGene00011639 | ztf-17 | T09A5.12 |
| WBGene00011651 | nhr-217 | T09E11.2 |
| WBGene00011661 | ztf-27 | T09F3.1 |
| WBGene00011696 | eea-1 | T10G3.5 |
| WBGene00011722 |  | T11G6.8 |
| WBGene00011729 | set-16 | T12D8.1 |
| WBGene00011743 |  | T13F2.2 |
| WBGene00011750 | nhr-218 | T13F3.2 |
| WBGene00011814 | gtf-2H2C | T16H12.4 |
| WBGene00011824 |  | T18D3.7 |
| WBGene00011924 |  | T22C8.3 |
| WBGene00011925 |  | T22C8.4 |
| WBGene00011926 | sptf-2 | T22C8.5 |
| WBGene00011956 |  | T23F11.4 |
| WBGene00011964 | saeg-2 | T23G5.6 |
| WBGene00012000 |  | T24H10.1 |
| WBGene00012005 | jun-1 | T24H10.7 |
| WBGene00012050 | nhr-223 | T26E4.8 |
| WBGene00012056 | nhr-285 | T26E4.16 |
| WBGene00012074 |  | T27A8.2 |
| WBGene00012101 | zip-10 | T27F2.4 |
| WBGene00012209 | hmg-20 | W02D9.3 |
| WBGene00012243 |  | W04D2.4 |
| WBGene00012277 | ccch-3 | W05B10.2 |
| WBGene00012317 | ztf-6 | W06H12.1 |
| WBGene00012330 | zip-3 | W07G1.3 |
| WBGene00012385 |  | Y5F2A.4 |
| WBGene00012405 | ztf-25 | Y6G8.3 |
| WBGene00012435 | flh-1 | Y11D7A.12 |
| WBGene00012436 | flh-3 | Y11D7A.13 |
| WBGene00012446 | nhr-230 | Y17D7A.1 |
| WBGene00012449 | nhr-231 | Y17D7B.1 |
| WBGene00012473 |  | Y17G7B.22 |
| WBGene00012474 | attf-6 | Y18D10A.1 |
| WBGene00012494 | nhr-232 | Y22F5A.1 |
| WBGene00012578 | ccct-1 | Y37H9A.3 |
| WBGene00012584 | ceh-100 | Y38E10A.6 |
| WBGene00012596 | nhr-234 | Y38E10A.18 |
| WBGene00012639 | fezf-1 | Y38H8A.5 |
| WBGene00012644 | rabx-5 | Y39A1A.5 |
| WBGene00012674 | bed-1 | Y39B6A.12 |
| WBGene00012694 | gtf-2F2 | Y39B6A.36 |
| WBGene00012702 | ztf-20 | Y39B6A.46 |
| WBGene00012703 | nhr-145 | Y39B6A.47 |
| WBGene00012715 | snpc-1.2 | Y39E4B.2 |
| WBGene00012718 | dhhc-8 | Y39E4B.7 |
| WBGene00012735 | sptf-3 | Y40B1A.4 |
| WBGene00012832 | dmd-3 | Y43F8C.10 |
| WBGene00012943 | bed-2 | Y47D3B.9 |
| WBGene00012948 | dhhc-2 | Y47H9C.2 |
| WBGene00012974 |  | Y48A6C.1 |
| WBGene00012976 | sup-35 | Y48A6C.3 |
| WBGene00012988 | ztf-22 | Y48C3A.4 |
| WBGene00012994 |  | Y48C3A.12 |
| WBGene00013006 | lin-38 | Y48E1B.7 |
| WBGene00013096 | mcd-1 | Y51H1A.6 |
| WBGene00013100 | zip-7 | Y51H4A.4 |
| WBGene00013111 | sta-1 | Y51H4A.17 |
| WBGene00013128 | dxbp-1 | Y52B11A.9 |
| WBGene00013134 | hsf-2 | Y53C10A.3 |
| WBGene00013147 | eyg-1 | Y53C12C.1 |
| WBGene00013150 |  | Y53F4B.3 |
| WBGene00013152 |  | Y53F4B.5 |
| WBGene00013178 |  | Y53H1A.2 |
| WBGene00013219 | elof-1 | Y54G11A.11 |
| WBGene00013225 |  | Y56A3A.2 |
| WBGene00013236 |  | Y56A3A.18 |
| WBGene00013240 |  | Y56A3A.28 |
| WBGene00013270 |  | Y57A10A.31 |
| WBGene00013319 | ccch-5 | Y57G11C.25 |
| WBGene00013350 | slr-2 | Y59A8B.13 |
| WBGene00013370 |  | Y60A9.3 |
| WBGene00013383 | aptf-2 | Y62E10A.17 |
| WBGene00013425 | ceh-91 | Y66A7A.5 |
| WBGene00013438 | ztf-29 | Y66D12A.12 |
| WBGene00013441 | xpb-1 | Y66D12A.15 |
| WBGene00013465 |  | Y67H2A.10 |
| WBGene00013483 | nhr-241 | Y69H2.8 |
| WBGene00013505 |  | Y71A12B.8 |
| WBGene00013512 | nhr-276 | Y71A12C.1 |
| WBGene00013529 | gft-2H4 | Y73F8A.24 |
| WBGene00013537 |  | Y73F8A.33 |
| WBGene00013560 | zip-12 | Y75B8A.29 |
| WBGene00013580 |  | Y79H2A.3 |
| WBGene00013583 | ceh-51 | Y80D3A.3 |
| WBGene00013584 | nhr-243 | Y80D3A.4 |
| WBGene00013595 | atg-4.1 | Y87G2A.3 |
| WBGene00013632 |  | Y105C5A.1 |
| WBGene00013639 |  | Y105C5A.15 |
| WBGene00013665 | hlh-32 | Y105C5B.29 |
| WBGene00013676 | ekl-4 | Y105E8A.17 |
| WBGene00013717 | madf-10 | Y106G6H.4 |
| WBGene00013734 |  | Y111B2A.10 |
| WBGene00013736 | gtf-2A2 | Y111B2A.13 |
| WBGene00013738 | elpc-2 | Y111B2A.17 |
| WBGene00013794 | dct-13 | Y116A8C.17 |
| WBGene00013795 | nhr-229 | Y116A8C.18 |
| WBGene00013796 |  | Y116A8C.19 |
| WBGene00013797 |  | Y116A8C.20 |
| WBGene00013799 | athp-3 | Y116A8C.22 |
| WBGene00013876 | ceh-74 | ZC376.4 |
| WBGene00013878 | atfs-1 | ZC376.7 |
| WBGene00013940 | nhr-248 | ZK218.6 |
| WBGene00013966 | ztf-9 | ZK287.6 |
| WBGene00013970 | klu-1 | ZK337.2 |
| WBGene00013976 | nhr-33 | ZK455.6 |
| WBGene00013998 | gtf-2E1 | ZK550.4 |
| WBGene00014060 |  | ZK673.4 |
| WBGene00014068 | nhr-255 | ZK678.2 |
| WBGene00014075 | dhhc-4 | ZK757.4 |
| WBGene00014114 | tftc-3 | ZK856.13 |
| WBGene00014131 | sdz-38 | ZK892.7 |
| WBGene00014189 | nhr-245 | ZK1025.10 |
| WBGene00014193 | nhr-247 | ZK1037.5 |
| WBGene00014208 |  | ZK1067.2 |
| WBGene00014230 | gtf-2H3 | ZK1128.4 |
| WBGene00015091 |  | B0261.1 |
| WBGene00015138 |  | B0310.2 |
| WBGene00015143 |  | B0336.3 |
| WBGene00015147 |  | B0336.7 |
| WBGene00015150 |  | B0336.13 |
| WBGene00015285 | gmeb-1 | C01B12.2 |
| WBGene00015296 | gtf-2F1 | C01F1.1 |
| WBGene00015352 |  | C02F5.12 |
| WBGene00015395 | nhr-147 | C03G6.8 |
| WBGene00015396 | nhr-148 | C03G6.10 |
| WBGene00015397 | nhr-149 | C03G6.12 |
| WBGene00015447 | srab-2 | C04F5.5 |
| WBGene00015451 |  | C04F5.9 |
| WBGene00015468 | madf-11 | C05D2.6 |
| WBGene00015477 | attf-4 | C05D10.1 |
| WBGene00015497 | nhr-76 | C05G6.1 |
| WBGene00015523 | ztf-30 | C06E1.8 |
| WBGene00015527 |  | C06E2.1 |
| WBGene00015555 | ufl-1 | C06G3.9 |
| WBGene00015620 |  | C08G9.2 |
| WBGene00015649 |  | C09F5.3 |
| WBGene00015651 | ceh-53 | C09G12.1 |
| WBGene00015705 | nhr-152 | C12D5.2 |
| WBGene00015716 | sox-4 | C12D12.5 |
| WBGene00015758 | nhr-155 | C14C6.4 |
| WBGene00015806 | smrc-1 | C16A3.1 |
| WBGene00015809 |  | C16A3.4 |
| WBGene00015813 | thoc-2 | C16A3.8 |
| WBGene00015869 | nhr-257 | C17A2.1 |
| WBGene00015897 | nhr-156 | C17E7.1 |
| WBGene00015900 | nhr-157 | C17E7.5 |
| WBGene00015901 | nhr-158 | C17E7.6 |
| WBGene00015902 | nhr-159 | C17E7.7 |
| WBGene00015934 | ceh-48 | C17H12.9 |
| WBGene00015971 | swsn-2.2 | C18E3.2 |
| WBGene00015981 | elt-7 | C18G1.2 |
| WBGene00016029 | elf-1 | C24A1.2 |
| WBGene00016091 | nhr-30 | C25E10.1 |
| WBGene00016126 | nhr-258 | C26B2.4 |
| WBGene00016138 | flh-2 | C26E6.2 |
| WBGene00016154 |  | C27A12.2 |
| WBGene00016162 | crh-2 | C27D6.4 |
| WBGene00016189 |  | C28G1.4 |
| WBGene00016200 | dpff-1 | C28H8.9 |
| WBGene00016220 |  | C29F9.5 |
| WBGene00016233 | srt-58 | C29G2.5 |
| WBGene00016364 | nhr-161 | C33G8.7 |
| WBGene00016365 | nhr-139 | C33G8.8 |
| WBGene00016366 | nhr-140 | C33G8.9 |
| WBGene00016367 | nhr-162 | C33G8.10 |
| WBGene00016368 | nhr-163 | C33G8.12 |
| WBGene00016383 | zgpa-1 | C33H5.17 |
| WBGene00016407 |  | C34D10.2 |
| WBGene00016427 |  | C34H4.5 |
| WBGene00016517 | nhr-260 | C38C3.9 |
| WBGene00016557 | ceh-84 | C40D2.4 |
| WBGene00016562 |  | C41D11.3 |
| WBGene00016600 | ets-5 | C42D8.4 |
| WBGene00016620 | dhhc-9 | C43H6.7 |
| WBGene00016712 |  | C46E10.8 |
| WBGene00016713 |  | C46E10.9 |
| WBGene00016754 | cebp-2 | C48E7.11 |
| WBGene00016772 | nhr-166 | C49D10.2 |
| WBGene00016777 | nhr-261 | C49D10.9 |
| WBGene00016798 | ets-8 | C50A2.4 |
| WBGene00016865 | ets-9 | C52B9.2 |
| WBGene00016870 | ets-10 | C52B9.11 |
| WBGene00016888 |  | C52E12.1 |
| WBGene00016890 | lst-5 | C52E12.6 |
| WBGene00016905 | ztf-3 | C53D5.4 |
| WBGene00016926 | nhr-171 | C54F6.8 |
| WBGene00016927 | nhr-172 | C54F6.9 |
| WBGene00016930 | madf-6 | C54G6.1 |
| WBGene00016948 | scrt-1 | C55C2.1 |
| WBGene00016975 | nhr-173 | C56E10.1 |
| WBGene00016997 | cebp-1 | D1005.3 |
| WBGene00017011 | eaf-1 | D1007.16 |
| WBGene00017075 | nap-1 | D2096.8 |
| WBGene00017198 | nhr-36 | F07C3.10 |
| WBGene00017317 | attf-2 | F09G2.9 |
| WBGene00017326 | dmd-5 | F10C1.5 |
| WBGene00017352 |  | F10E7.11 |
| WBGene00017406 | sdz-12 | F12E12.5 |
| WBGene00017423 |  | F13C5.2 |
| WBGene00017430 | bcl-11 | F13H6.1 |
| WBGene00017482 | set-9 | F15E6.1 |
| WBGene00017503 | nhr-177 | F16B4.1 |
| WBGene00017510 | nhr-178 | F16B4.9 |
| WBGene00017512 | nhr-179 | F16B4.11 |
| WBGene00017535 | atf-8 | F17A9.3 |
| WBGene00017538 | ceh-49 | F17A9.6 |
| WBGene00017598 |  | F19F10.1 |
| WBGene00017601 | ets-7 | F19F10.5 |
| WBGene00017606 | ets-6 | F19F10.10 |
| WBGene00017651 |  | F21A9.2 |
| WBGene00017664 | npax-1 | F21D12.5 |
| WBGene00017687 | ets-4 | F22A3.1 |
| WBGene00017690 | ceh-60 | F22A3.5 |
| WBGene00017733 | ubxn-1 | F23C8.4 |
| WBGene00017742 | nfyc-1 | F23F1.1 |
| WBGene00017755 | zip-8 | F23F12.9 |
| WBGene00017758 |  | F23H11.2 |
| WBGene00017787 | nhr-141 | F25E5.6 |
| WBGene00017814 |  | F26A10.2 |
| WBGene00017961 | nhr-180 | F31F4.12 |
| WBGene00017967 | ada-2 | F32A5.1 |
| WBGene00018009 | dhhc-3 | F33D11.12 |
| WBGene00018022 | ceh-87 | F34D6.2 |
| WBGene00018099 | ztf-28 | F36F12.8 |
| WBGene00018140 |  | F37B4.10 |
| WBGene00018189 | nhr-181 | F38H12.3 |
| WBGene00018248 |  | F40G9.14 |
| WBGene00018265 | nhr-182 | F41B5.9 |
| WBGene00018266 | nhr-183 | F41B5.10 |
| WBGene00018355 | ceh-86 | F42G2.6 |
| WBGene00018391 | rpb-4 | F43E2.2 |
| WBGene00018404 | nhr-39 | F44A2.4 |
| WBGene00018412 | nhr-37 | F44C4.2 |
| WBGene00018415 | nhr-184 | F44C8.9 |
| WBGene00018420 |  | F44E2.7 |
| WBGene00018430 | nhr-142 | F44E7.8 |
| WBGene00018432 | irld-33 | F45C12.1 |
| WBGene00018433 | ceh-82 | F45C12.2 |
| WBGene00018434 | ceh-81 | F45C12.3 |
| WBGene00018446 | ceh-83 | F45C12.15 |
| WBGene00018539 | nhr-185 | F47C10.1 |
| WBGene00018541 | nhr-186 | F47C10.3 |
| WBGene00018542 | nhr-187 | F47C10.4 |
| WBGene00018544 | nhr-188 | F47C10.7 |
| WBGene00018545 | nhr-189 | F47C10.8 |
| WBGene00018567 |  | F47E1.3 |
| WBGene00018591 | npax-2 | F48B9.5 |
| WBGene00018622 | nhr-190 | F48G7.11 |
| WBGene00018636 |  | F49E8.2 |
| WBGene00018704 | ztf-13 | F52E4.8 |
| WBGene00018740 | tra-4 | F53B3.1 |
| WBGene00018769 | mnat-1 | F53G2.7 |
| WBGene00018786 | hmbx-1 | F54A5.1 |
| WBGene00018794 | attf-3 | F54C4.3 |
| WBGene00018833 | ztf-1 | F54F2.5 |
| WBGene00018836 |  | F54F2.9 |
| WBGene00018892 | cir-1 | F55F8.4 |
| WBGene00018959 |  | F56D1.1 |
| WBGene00018990 | klf-1 | F56F11.3 |
| WBGene00018993 | nhr-266 | F56H1.2 |
| WBGene00019011 |  | F57C9.4 |
| WBGene00019115 | nhr-195 | F59E11.10 |
| WBGene00019116 | nhr-143 | F59E11.11 |
| WBGene00019137 | ceh-85 | F59H6.6 |
| WBGene00019217 | athp-2 | H20J04.2 |
| WBGene00019218 | madf-3 | H20J04.3 |
| WBGene00019257 | dhhc-13 | H32C10.3 |
| WBGene00019299 | snai-1 | K02D7.2 |
| WBGene00019327 | zip-2 | K02F3.4 |
| WBGene00019344 | dhhc-10 | K02G10.1 |
| WBGene00019347 | mbl-1 | K02H8.1 |
| WBGene00019380 | K04C2.2 | K04C2.2 |
| WBGene00019407 |  | K05F1.5 |
| WBGene00019424 | aptf-1 | K06A1.1 |
| WBGene00019521 | dmd-7 | K08B12.2 |
| WBGene00019598 |  | K09H9.7 |
| WBGene00019611 |  | K10B3.5 |
| WBGene00019629 | cid-1 | K10D2.3 |
| WBGene00019651 |  | K11D12.12 |
| WBGene00019691 |  | K12H6.12 |
| WBGene00019741 | nhr-201 | M02H5.3 |
| WBGene00019742 | nhr-202 | M02H5.4 |
| WBGene00019743 | nhr-203 | M02H5.5 |
| WBGene00019751 |  | M03D4.4 |
| WBGene00019816 | nhr-204 | R02C2.4 |
| WBGene00019821 | gtf-2H1 | R02D3.3 |
| WBGene00019824 |  | R02D3.7 |
| WBGene00019864 | ceh-93 | R04A9.5 |
| WBGene00019878 |  | R05D3.3 |
| WBGene00019960 | ztf-16 | R08E3.4 |
| WBGene00020015 | nhr-210 | R11G11.12 |
| WBGene00020062 | nhr-270 | R13D11.8 |
| WBGene00020066 | dhhc-5 | R13F6.5 |
| WBGene00020093 |  | R144.3 |
| WBGene00020111 |  | R151.8 |
| WBGene00020152 | nhr-211 | T01G6.5 |
| WBGene00020153 | nhr-212 | T01G6.6 |
| WBGene00020172 | thoc-1 | T02H6.2 |
| WBGene00020189 | tfbm-1 | T03F1.7 |
| WBGene00020214 | attf-5 | T04G9.1 |
| WBGene00020251 | camt-1 | T05C1.4 |
| WBGene00020320 |  | T07F8.4 |
| WBGene00020368 | ast-1 | T08H4.3 |
| WBGene00020385 | nhr-216 | T09D3.4 |
| WBGene00020399 | ztf-4 | T10B11.3 |
| WBGene00020408 |  | T10D4.6 |
| WBGene00020460 | nhr-273 | T12C9.1 |
| WBGene00020480 | ssup-72 | T13C2.4 |
| WBGene00020485 | ceh-54 | T13C5.4 |
| WBGene00020555 | nhr-219 | T19A5.5 |
| WBGene00020591 | nhr-220 | T19H12.8 |
| WBGene00020630 |  | T20F7.1 |
| WBGene00020635 |  | T20H4.2 |
| WBGene00020694 | dhhc-11 | T22E7.2 |
| WBGene00020708 | dmd-8 | T22H9.4 |
| WBGene00020748 | nhr-221 | T24A6.8 |
| WBGene00020750 | nhr-222 | T24A6.11 |
| WBGene00020763 | ztf-18 | T24C4.7 |
| WBGene00020779 |  | T24G10.2 |
| WBGene00020823 |  | T26A5.8 |
| WBGene00020827 |  | T26A8.4 |
| WBGene00020849 | nhr-225 | T27B7.2 |
| WBGene00020850 | nhr-226 | T27B7.3 |
| WBGene00020851 | nhr-227 | T27B7.5 |
| WBGene00020852 | nhr-228 | T27B7.6 |
| WBGene00020930 | hlh-30 | W02C12.3 |
| WBGene00020942 |  | W02D7.6 |
| WBGene00020961 | sknr-1 | W02H5.7 |
| WBGene00021000 |  | W03F9.2 |
| WBGene00021019 |  | W04B5.2 |
| WBGene00021047 | zfp-3 | W05H7.4 |
| WBGene00021082 | zip-11 | W08E12.1 |
| WBGene00021132 | nfyb-1 | W10D9.4 |
| WBGene00021163 | nhr-275 | Y5H2A.2 |
| WBGene00021237 | crtc-1 | Y20F4.2 |
| WBGene00021254 |  | Y22D7AL.16 |
| WBGene00021281 | ell-1 | Y24D9A.1 |
| WBGene00021311 | thoc-5 | Y32H12A.2 |
| WBGene00021363 | taf-6.2 | Y37E11AL.8 |
| WBGene00021374 |  | Y37E11B.1 |
| WBGene00021387 |  | Y37F4.6 |
| WBGene00021417 | nhr-236 | Y38F2AL.5 |
| WBGene00021423 | tftc-1 | Y38F2AR.5 |
| WBGene00021446 | hlh-33 | Y39A3CR.6 |
| WBGene00021522 | nhr-274 | Y41D4B.21 |
| WBGene00021538 | rabs-5 | Y42H9AR.3 |
| WBGene00021552 | zip-4 | Y44E3B.1 |
| WBGene00021610 | nhr-237 | Y46H3D.6 |
| WBGene00021611 | nhr-238 | Y46H3D.7 |
| WBGene00021636 | pcaf-1 | Y47G6A.6 |
| WBGene00021637 |  | Y47G6A.7 |
| WBGene00021689 |  | Y48G8AL.10 |
| WBGene00021704 |  | Y48G9A.11 |
| WBGene00021714 | cyh-1 | Y49F6B.1 |
| WBGene00021812 |  | Y53G8AR.5 |
| WBGene00021816 |  | Y53G8AR.9 |
| WBGene00021830 |  | Y54E10A.10 |
| WBGene00021845 | rpb-7 | Y54E10BR.6 |
| WBGene00021846 | ztf-23 | Y54E10BR.8 |
| WBGene00021848 | nhr-239 | Y54F10AM.1 |
| WBGene00021885 |  | Y54G2A.20 |
| WBGene00021904 | gtf-2H5 | Y55B1AL.2 |
| WBGene00021924 |  | Y55F3AM.6 |
| WBGene00021931 |  | Y55F3AM.14 |
| WBGene00021942 | madf-1 | Y55F3BR.5 |
| WBGene00022000 | tbx-42 | Y59E9AR.5 |
| WBGene00022060 | dmd-9 | Y67D8A.3 |
| WBGene00022097 | nhr-242 | Y69A2AR.26 |
| WBGene00022182 | swsn-3 | Y71H2AM.17 |
| WBGene00022207 | elk-2 | Y73B3A.5 |
| WBGene00022278 | rcor-1 | Y74C9A.4 |
| WBGene00022334 |  | Y82E9BR.1 |
| WBGene00022349 |  | Y82E9BR.17 |
| WBGene00022374 | nhr-277 | Y94H6A.1 |
| WBGene00022423 | nhr-41 | Y104H12A.1 |
| WBGene00022500 | lfi-1 | ZC8.4 |
| WBGene00022518 | zfh-2 | ZC123.3 |
| WBGene00022554 | duxl-1 | ZC204.2 |
| WBGene00022592 | klu-2 | ZC328.2 |
| WBGene00022598 | ztf-8 | ZC395.8 |
| WBGene00022608 | madf-9 | ZC416.1 |
| WBGene00022636 | nhr-278 | ZK6.1 |
| WBGene00022637 | nhr-252 | ZK6.2 |
| WBGene00022639 | nhr-253 | ZK6.4 |
| WBGene00022640 | nhr-254 | ZK6.5 |
| WBGene00022671 |  | ZK177.3 |
| WBGene00022681 |  | ZK185.1 |
| WBGene00022695 |  | ZK328.6 |
| WBGene00022755 | nhr-250 | ZK488.1 |
| WBGene00022756 | nhr-251 | ZK488.4 |
| WBGene00022762 |  | ZK546.5 |
| WBGene00022785 |  | ZK652.6 |
| WBGene00022794 | snu-23 | ZK686.4 |
| WBGene00022795 |  | ZK686.5 |
| WBGene00022805 | nhr-256 | ZK697.2 |
| WBGene00022837 | ceh-45 | ZK993.1 |
| WBGene00022861 | dve-1 | ZK1193.5 |
| WBGene00023497 | lin-15B | ZK662.4 |
| WBGene00043056 | nfya-2 | Y53H1A.5 |
| WBGene00043705 | egrh-2 | Y55F3AM.7 |
| WBGene00044032 | ceh-99 | T21B4.17 |
| WBGene00044068 | syd-9 | ZK867.1 |
| WBGene00044071 | dhhc-14 | D2021.2 |
| WBGene00044072 | ham-3 | ZK1128.5 |
| WBGene00044078 | tag-243 | T04A8.4 |
| WBGene00044330 | alr-1 | R08B4.2 |
| WBGene00044386 |  | C27A2.7 |
| WBGene00044508 | nsy-7 | C18F3.4 |
| WBGene00044699 | nhr-286 | VC5.6 |
| WBGene00044791 | bnc-1 | F55C5.11 |
| WBGene00044798 | tbx-43 | Y46E12A.4 |
| WBGene00044805 |  | Y53C10A.15 |
| WBGene00045215 | ceh-63 | C02F12.10 |
| WBGene00045255 | nhr-146 | Y41D4B.27 |
| WBGene00045515 |  | ZK1037.13 |
| WBGene00077521 | maf-1 | F45H11.6 |
| WBGene00077761 | zip-9 | F17C11.17 |
