# Supplementary material for: Wild-type Caenorhabditis elegans isolates exhibit distinct gene expression profiles in response to microbial infection
Source: BMC Genomics. 2022 Mar 23;23:229. doi: 10.1186/s12864-022-08455-2 (PMC8943956; doi:10.1186/s12864-022-08455-2)
Supplement: Supplementary file 2 — Additional file 2. [file 12864_2022_8455_MOESM2_ESM.docx]

| **Supplemental Table 1: Median survival of pathogen susceptibility experiments** | | | |
| --- | --- | --- | --- |
| Experiment | Trial | Total worms | Median survival (days) |
| *S. epidermidis* (EVL 2000) |  |  |  |
| N2 | 1 | 30 | 22 |
| N2 | 2 | 30 | 22 |
| N2 | 3 | 30 | 19 |
| N2 | 4 | 30 | 15 |
| N2 | 5 | 30 | 16 |
|  |  |  |  |
| CB4856 | 1 | 30 | 8 |
| CB4856 | 2 | 30 | 16 |
| CB4856 | 3 | 30 | 13 |
| CB4856 | 4 | 30 | 10 |
| CB4856 | 5 | 30 | 6 |
| CB4856 | 6 | 30 | 15 |
|  |  |  |  |
| *S. epidermidis* (ATCC 700562) |  |  |  |
| N2 | 1 | 30 | 11 |
| N2 | 2 | 30 | 12 |
| N2 | 3 | 30 | 14 |
|  |  |  |  |
| CB4856 | 1 | 30 | 4 |
| CB4856 | 2 | 30 | 5 |
| CB4856 | 3 | 30 | 8 |
|  |  |  |  |
| *P. aeruginosa* (PA-14) |  |  |  |
| N2 | 1 | 30 | 4 |
| N2 | 2 | 30 | 4 |
| N2 | 3 | 30 | 4 |
| N2 | 4 | 30 | 3 |
|  |  |  |  |
| CB4856 | 1 | 30 | 3 |
| CB4856 | 2 | 30 | 4 |
| CB4856 | 3 | 30 | 4 |
| CB4856 | 4 | 30 | 3 |
|  |  |  |  |
| *P. rettgeri* (Dmel1) |  |  |  |
| N2 | 1 | 30 | 10 |
| N2 | 2 | 30 | 8 |
| N2 | 3 | 30 | 8 |
| N2 | 4 | 30 | 6 |
| N2 | 5 | 30 | 7 |
|  |  |  |  |
| CB4856 | 1 | 30 | 7 |
| CB4856 | 2 | 30 | 8 |
| CB4856 | 3 | 30 | 8 |
| CB4856 | 4 | 30 | 5 |
| CB4856 | 5 | 30 | 8 |
|  |  |  |  |
| *S. aureus* (Newman) |  |  |  |
| N2 | 1 | 30 | 8 |
| N2 | 2 | 30 | 9 |
| N2 | 3 | 30 | 3 |
| N2 | 4 | 30 | 8 |
|  |  |  |  |
| CB4856 | 1 | 30 | 9 |
| CB4856 | 2 | 30 | 7 |
| CB4856 | 3 | 30 | 4 |
| CB4856 | 4 | 30 | 8 |
|  |  |  |  |
| *E. coli* OP50 |  |  |  |
| N2 | 1 | 30 | 11 |
| N2 | 2 | 30 | 11 |
| N2 | 3 | 30 | 11 |
| N2 | 4 | 30 | 13 |
| N2 | 5 | 30 | 13 |
| N2 | 6 | 30 | 12 |
| N2 | 7 | 30 | 13 |
|  |  |  |  |
| CB4856 | 1 | 30 | 16 |
| CB4856 | 2 | 30 | 15 |
| CB4856 | 3 | 30 | 16 |
| CB4856 | 4 | 30 | 16 |
| CB4856 | 5 | 30 | 15 |
| CB4856 | 6 | 30 | 19 |
|  |  |  |  |
|  |  |  |  |
